# Inhibition of RIPK1 by ZJU-37 promotes oligodendrocyte progenitor proliferation and remyelination via NF-κB pathway

**DOI:** 10.1038/s41420-022-00929-2

**Published:** 2022-04-01

**Authors:** Xiao-Ru Ma, Shu-Ying Yang, Shuang-Shuang Zheng, Huan-Huan Yan, Hui-Min Gu, Fan Wang, Yang Wu, Zhao-Jun Dong, Di-Xian Wang, Yue Wang, Xianhui Meng, Jie Sun, Hong-Guang Xia, Jing-Wei Zhao

**Affiliations:** 1 Department of Pathology and Department of Human Anatomy, Histology and Embryology of Sir Run Run Shaw Hospital, System Medicine Research Center, NHC and CAMS Key Laboratory of Medical Neurobiology, Zhejiang University School of Medicine, Hangzhou, 310058 China; 2 Department of Biochemistry & Research Center of Clinical Pharmacy of The First Affiliated Hospital, Zhejiang University School of Medicine, Hangzhou, 310058 China; 3 Bone Marrow Transplantation Center of the First Affiliated Hospital, and Department of Cell Biology, Zhejiang University School of Medicine, Hangzhou, 310058 China; 4 Liangzhu Laboratory, Zhejiang University Medical Center, Hangzhou, 311121 China; 5 Center of Cryo-Electron Microscopy, Zhejiang University, Hangzhou, 310058 China

**Keywords:** Oligodendrocyte, Multiple sclerosis, Cell death in the nervous system

## Abstract

Receptor interacting serine/threonine protein kinase 1 (RIPK1) activation and necroptosis have been genetically and mechanistically linked with human multiple sclerosis and neurodegenerative diseases for which demyelination is a common key pathology. Demyelination can be healed through remyelination which is mediated by new oligodendrocytes derived from the adult oligodendrocyte progenitor cells (OPCs). Unfortunately, the efficiency of remyelination declines with progressive aging partially due to the depletion of OPCs following chronic or repeated demyelination. However, to our knowledge, so far there is no drug which enhances proliferation of OPCs, and it is unknown whether inhibiting RIPK1 activity directly affect OPCs, the central player of remyelination. Using TNFα induced RIPK1-dependent necroptosis in Jurkat FADD^−/−^ cells as a cell death assay, we screened from 2112 FDA-approved drugs and the drug candidates of new RIPK1 inhibitors selected by ourselves, and identified ZJU-37, a small molecule modified by introducing an amide bond to Nec-1s, is a new RIPK1 kinase inhibitor with higher potency than Nec-1s which has the best reported potency. We unveil in addition to protecting myelin from demyelination and axons from degeneration, ZJU-37 exhibits a new role on promoting proliferation of OPCs and enhancing remyelination by inhibiting RIPK1 kinase activity with higher potency than Nec-1s. Mechanistically, ZJU-37 promotes proliferation of OPCs by enhancing the transcription of platelet derived growth factor receptor alpha via NF-κB pathway. This work identifies ZJU-37 as a new drug candidate which enhances remyelination by promoting proliferation of OPCs, paving the way for a potential drug to enhance myelin repair.

## Introduction

Demyelination, axonal degeneration and neuroinflammation are key pathology of multiple sclerosis (MS), an autoimmune disease of the central nervous system (CNS) [1, 2] with autoimmune-mediated demyelination as the core pathology [1–3]. After demyelination, a spontaneous regenerative process called remyelination is triggered, and the oligodendrocyte progenitor cells (OPCs) are responsible for this process which limits and repairs the damage [3]. Unfortunately, the efficiency of remyelination declines in many MS patients especially those in the progressive phase, largely due to both the declined intrinsic capacity of OPCs and the deteriorated pro-inflammatory microenvironment [3, 4]. The density of OPCs within the CNS lesions of MS patients becomes less than the non-lesioned surrounding [5], and such reduction exacerbates further with progressive aging [6], highlighting the necessity to expand the pool size of the adult OPCs. Targeting its key pathology, there have been different drugs and ongoing clinical trials on MS [4, 7], however, none of them enhances proliferation of OPCs. Therefore, searching new drug candidate which promotes proliferation of OPCs and remyelination is of great significance for the treatment of MS.

Accumulating evidence links MS with activation of necroptosis. The level of tumor necrosis factor alpha (TNFα), the ligand for necroptosis, is elevated in the serum, brain and cerebral spinal fluid of MS patients, and the hallmark mediators of necroptosis including receptor interacting serine/threonine protein kinase 1 (RIPK1) are activated in both mature oligodendrocytes [8] and neurons [9] within demyelinated lesion of both animal models and MS patients. Importantly, inhibiting necroptosis by Nec-1s, so far the most potent inhibitor of RIPK1 activity [10–12], protects mature oligodendrocytes from death [8, 13] and attenuates symptoms in a MS mouse model [14], indicating that inhibiting RIPK1 activity both protects myelin from demyelination and reduces neuroinflammation [8, 9, 14]. However, it is unknown whether inhibiting RIPK1 activity directly affect OPCs, the central player of remyelination. Due to its great potential as a drug candidate, it is of great value to develop new potent RIPK1 inhibitors.

In this study, we identified ZJU-37, a modified form of Nec-1s by introducing an amide bond, as a new RIPK1 kinase inhibitor with higher potency than Nec-1s and further uncovered a new role of ZJU-37 on promoting proliferation of OPCs and enhancing remyelination. Our results unveil that ZJU-37 is a potential drug candidate for demyelinating diseases.

## Results

### A high-throughput screen identifies ZJU-37 as a RIPK1 kinase inhibitor with higher potency than Nec-1s

**Fig. 1:**
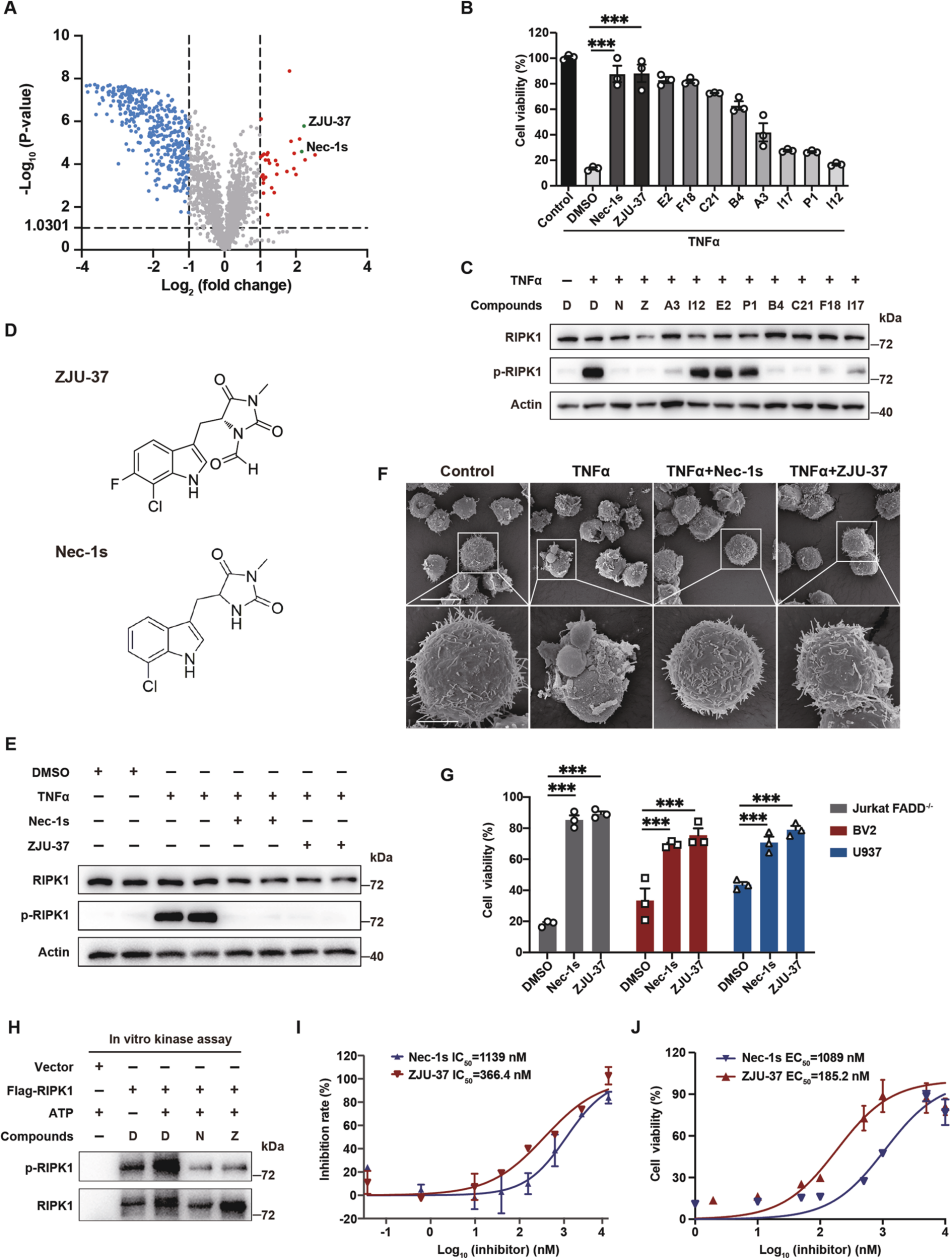
A high-throughput screen identifies ZJU-37 as a RIPK1 kinase inhibitor with higher potency than Nec-1s. **A** High-throughput screening of a FDA approved 2112 compounds library and ZJU-37, a modified small-molecule compound of Nec-1 produced by ourselves, in Jurkat FADD^−/−^ cells by inducing necroptosis. From this compound library, 32 compounds including Nec-1s and ZJU-37 showed protective effect from cell necroptosis. Concentration of compounds: 10 μM. “fold” refers to “the ratio of the cell viability of TNFα + compound treated Jurkat FADD^−/−^ cells to that of TNFα + DMSO treated Jurkat FADD^−/−^ cells”. **B** Quantification of cell viability in TNFα ± indicated compounds-treated Jurkat FADD^−/−^ cells (one-way ANOVA, Tukey’s post hoc test, *n* = 3 plate wells for each compound). E2: Debriefing; F18: Pexmetinib; C21: GSK2606414; B4: GSK2656157; A3: Nintedanib; I17: TAK-632; P1: Pazopanib; I12: Osthole. **C** Western blot of RIPK1 and (p-RIPK1) in Jurkat FADD^−/−^ cells (D: DMSO; N: Nec-1s; Z: ZJU-37). **D** The structure of ZJU-37 and Nec-1s. **E** Jurkat FADD^−/−^ cells treated with various combinations of ± TNFα, ± Nec-1s, and ±ZJU-37. The levels of RIPK1 and p-RIPK1 were determined by western blot. **F** Representative images of scanning electron microscope showing necroptosis induced by TNFα in Jurkat FADD^−/−^ cells and protected by Nec-1s and ZJU-37. Scale bars, 20 μm (top row), 5 μm (bottom row). **G** Quantification of cell viability in Jurkat FADD^−/−^ (TNFα), BV2 (z-VAD), or U937 (TNFα and z-VAD) cells after treatment as indicated (two-way repeated ANOVA, Sidak’s post hoc test, *n* = 3 plate wells for each compound). **H** In vitro kinase assay. HEK293T cells were transfected with Flag-RIPK1 or vehicle and treated with Nec-1s or ZJU-37 for 20 h. Flag-RIPK1 was immunoprecipitated using anti-Flag and incubated with or without 50 μM inhibitors as indicated at 30 °C for 30 min. The levels of RIPK1 and p-RIPK1 were determined by western blot. **I** Inhibitory effectiveness of RIPK1 kinase activity comparison between Nec-1s and ZJU-37 in the kinase-Glo assay (in vitro kinase assay shown in H). 8 different concentrations of both Nec-1s and ZJU-37 were used in this experiment (*n* = 2 plate wells). **J** Protective potency from necroptosis of Jurkat FADD^−/−^ cells treated with TNFα ± Nec-1s or TNFα ± ZJU-37 was measured using CellTiter-Glo assay. 8 different concentrations of both Nec-1s and ZJU-37 were used in this experiment (*n* = 3 plate wells). All data are presented as mean ± SEM. *P < 0.05; **P < 0.01, ***P < 0.001, n.s.: no significance.

RIPK1 is a critical regulator of necroptosis [12], and in Jurkat FADD^−/−^ cells TNFα can induce RIPK1-dependent necroptosis [15, 16] which is notably protected by necrostatin-1s (Nec-1s), a RIPK1 kinase activity inhibitor with the best reported potency [10–12]. Using this cell death assay based high-throughput screen model, we screened from 2112 FDA-approved drugs and the drug candidates of new RIPK1 inhibitors selected by ourselves (Fig. 1A). We found that among the top 9 positive hits, the cell viability is best rescued by ZJU-37, a small-molecule compound modified from Nec-1s and was produced in our lab, exhibited potency comparable to Nec-1s at higher dose (Fig. 1B). To determine whether the identified hits are RIPK1 kinase inhibitors, we tested the level of p-RIPK1 (S166), the hallmark of RIPK1 kinase activation. We found that the level of p-RIPK1 decreased to different levels after the treatment of ZJU-37 and other hits (Fig. 1C). Interestingly, both ZJU-37 and Nec-1s show the best potency in inhibiting RIPK1 activity among all the hits tested (Fig. 1C–E and Supplementary Table 1). Our screen also revealed a series of novel RIPK1 inhibitors including several drug candidates which are undergoing clinical trials and have not reported previously (Supplementary Table 2). To observe the protective effect of ZJU-37 and Nec-1s on the cell morphology change caused by TNFα induced necroptosis, using scanning electron microscope (SEM), we first established the typical cell surface features of TNFα induced necroptosis: reduction or loss of microvilli; plasma membrane shrinking, breaking down or forming cluster of small bulbs (Fig. 1F). We confirmed that both ZJU-37 and Nec-1s effectively protected Jurkat FADD^−/−^ cells from necroptosis (Fig. 1F). Consistently, we found that ZJU-37 strongly inhibited necroptosis in several different cell lines, including Jurkat FADD^−/−^, BV2 and U937 cells (Fig. 1G). By using an in vitro kinase assay, we confirmed that ZJU-37 inhibits the kinase activity of RIPK1 indeed (Fig. 1H). Comparison of the potency and titer between ZJU-37 and Nec-1s showed that the IC_50_ of ZJU-37 inhibiting RIPK1 kinase activity in vitro was 366.4 nM, less than one third of Nec-1s (Fig. 1I). Consistently, the EC_50_ of ZJU-37 for cell viability in Jurkat FADD^−/−^ cells was less than one fifth of that for Nec-1s (Fig. 1J). These results identify ZJU-37 as a RIPK1 kinase inhibitor which exhibits higher potency than Nec-1s.

### ZJU-37 exhibits dual protective effects in vivo

**Fig. 2:**
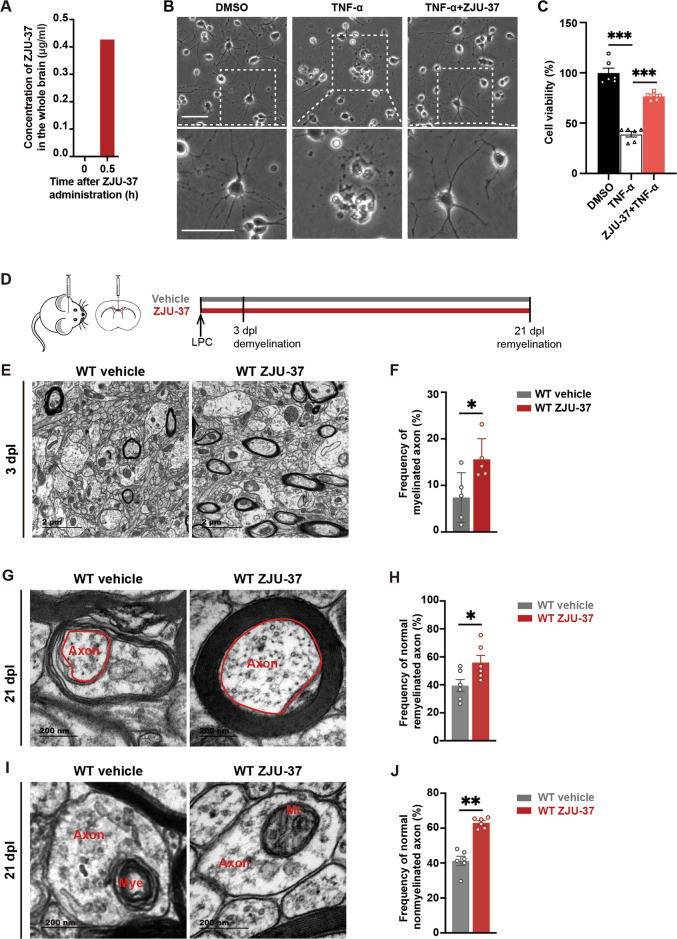
ZJU-37 exhibits dual protective effects in vivo. **A** ZJU-37 was measured in the supernatant of the whole brain by HPLC 30 min after intraperitoneal injection of ZJU-37 (4 mg/kg). **B** Representative images of rat OPCs treated with mouse TNF-α (100 ng/ml) ± ZJU-37 (5 μM) for 36 h. Scale bar: 50 μm. **C** Quantification of cell viability in TNF-α ± ZJU-37 treated OPCs (one-way ANOVA, Tukey’s post hoc test, *n* = 6 plate wells for each group). **D** Experimental design to evaluate demyelination and the ultrastructure of axons in the demyelination lesion. **E** Representative transmission electron micrograph within the demyelinated lesion at 3 dpl. **F** Quantification of the myelinated axons (two-tailed *t* test) within the demyelination region at 3 dpl (*n* = 5 mice). **G** Representative transmission electron micrographs of axons that were wrapped with myelin within the demyelination region at 21 dpl. **H** Quantification of the axons with normal ultrastructure and with myelin (two-tailed *t* test) within the demyelination region at 21 dpl (*n* = 6 mice). Scale bars, 1 μm. **I** Representative transmission electron micrographs of non-myelinated axons within the demyelination region at 21 dpl. Mye: myelin, Mt: mitochondria. **J** Quantification of the axons with normal ultrastructure but without myelin (two-tailed *t* test) within the demyelination region at 21 dpl (*n* = 6 mice). All data are presented as mean ± SEM. *P < 0.05, **P < 0.01, ***P < 0.001, n.s. no significance.

Next we found that ZJU-37 penetrated the blood-brain barrier (BBB) and entered into the brain parenchyma (Fig. 2A). There was no significant bodyweight change (Supplementary Fig. 1) and no abnormal appearance in the ZJU-37 group. Taking previous reports into account [12], our preliminary data suggest that no obvious toxicity was caused by ZJU-37.

Inhibition of RIPK1 can effectively ameliorate the pathologies and promote remyelination in various neurodegenerative diseases including MS [8, 17] and amyotrophic lateral sclerosis (ALS) [17, 18] mainly mediated by protecting oligodendrocytes from necroptosis [8, 13]. To further explore whether ZJU-37 protects OPCs from necroptosis in vitro, we induced OPC necroptosis by adding mouse TNF-α (Fig. 2B, C), and found the death of OPCs was rescued by ZJU-37 (Fig. 2B, C), indicating that ZJU-37 protects OPCs from TNF-α induced necroptosis in vitro. To explore whether ZJU-37 can protect myelin from demyelination in vivo, we induced demyelination in the mouse corpus callosum by focal injection of lysolecithin (LPC), a well-established in vivo model for studying demyelination and remyelination [3]. Half an hour before the LPC injection, ZJU-37 or vehicle were intraperitoneally (i.p.) injected into the mice, and then ZJU-37 or vehicle was once daily injected for 3 days when demyelination has completed [19, 20] (Fig. 2D). Using transmission electron microscope (TEM), we showed that at 3 days post lesion (dpl) (Fig. 2E), the frequency of myelinated axons within the lesion in the ZJU-37 treatment group was doubled than that of the vehicle group (Fig. 2F), indicating that ZJU-37 protects myelin from demyelination even in a very toxic condition. Given that inhibition of RIPK1 can protect axons from degeneration [18, 21], using TEM we showed that at 21 dpl when remyelination completes, some axons in the control group shrank or collapsed (Fig. 2G), contained myelin spheres (Fig. 2I) and abnormal-looking mitochondria in the axon and the space between axon and myelin expanded (Fig. 2G). Quantification verified that the proportion of normal-looking axons either with myelin (Fig. 2H) or without myelin (Fig. 2J) is higher in the ZJU-37 treated group, confirming that ZJU-37 protects axons from degeneration even within toxin-induced demyelination lesion. These data confirmed the dual protective effects of ZJU-37 in vivo: protects myelin from demyelination and axons from degeneration.

### ZJU-37 promotes OPC proliferation in vitro with higher efficacy than Nec-1s

**Fig. 3:**
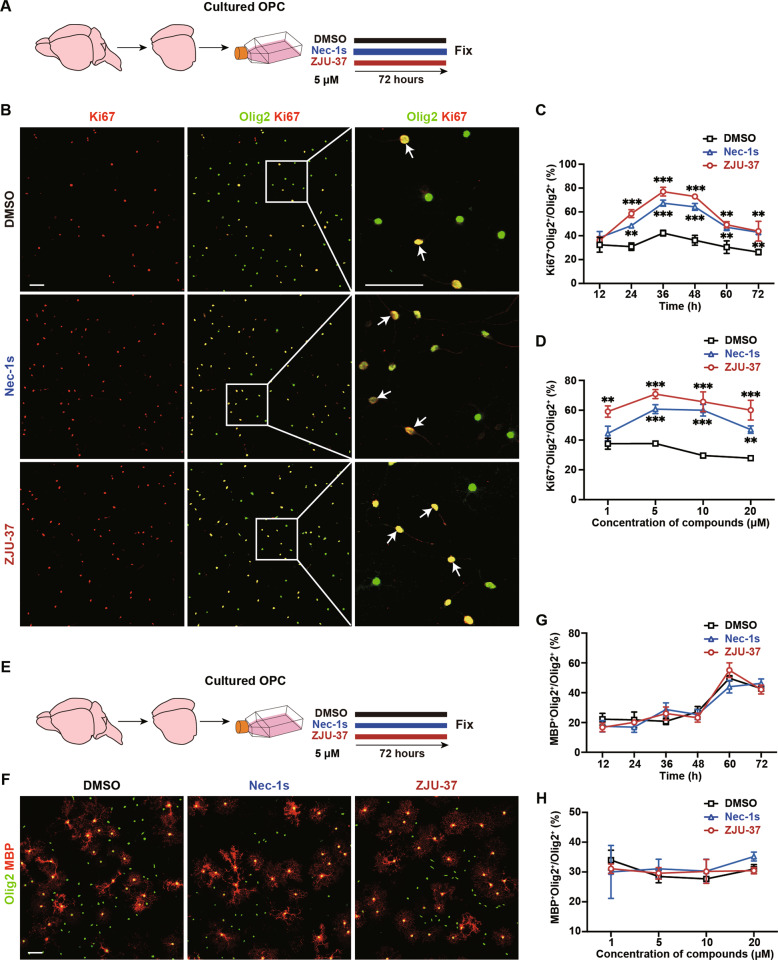
ZJU-37 promotes OPC proliferation in vitro with higher efficacy than Nec-1s. **A** Schematic diagram of the experiment for testing OPC proliferation in vitro. **B** Representative fluorescence images of proliferating primary rat OPCs treated with DMSO, Nec-1s (5 μM) or ZJU-37 (5 μM) for 36 h. Proliferating OPCs was visualized by Ki67 (red) and Olig2 (green). Scale bars, 50 μm. Quantification of proliferating OPCs over (**C**) time and (**D**) concentration of compounds (two-way repeated ANOVA, Sidak’s post hoc test, *n* = 3 plate wells). **E** Schematic diagram of the experiment for testing OPC differentiation in vitro. **F** Representative fluorescence images of differentiated primary rat OPCs treated with DMSO, Nec-1s or ZJU-37 for 48 h. Differentiated mature oligodendrocyte was visualized by MBP (red) and Olig2 (green). Scale bars, 50 μm. Quantification of differentiated OPC over (**G**) time and (**H**) drug concentration (two-way repeated ANOVA, Sidak’s post hoc test, *n* = 3 plate wells). All data are presented as mean ± SEM. *P < 0.05, **P < 0.01, ***P < 0.001, n.s. no significance.

We next explored whether ZJU-37 exhibits effects other than protection from necroptosis. It is unknown whether ZJU-37 affects OPCs, the adult neural stem cells responsible for remyelination [3, 22]. To this end, we purified OPCs from newborn rat brain and tested their proliferation (Fig. 3A, B) or differentiation (Fig. 3E, F) after compounds treatment. We found that after 5 μM ZJU-37 treatment, the proportion of proliferating OPCs was significantly higher than that of the DMSO group and peaked at 36 h (Fig. 3C). Nec-1s also promoted OPC proliferation, but to a lower level than ZJU-37 (Fig. 3C). Titer test with different concentrations revealed that 5 μM ZJU-37 promoted OPC proliferation most effectively (Fig. 3D). 5 μM Nec-1s also promoted OPC proliferation, but did not reach the level of ZJU-37 (Fig. 3D). However, neither ZJU-37 nor Nec-1s affected the differentiation of OPCs at all concentrations and treatment times tested (Fig. 3G, H). Our results indicated that ZJU-37 exhibits higher potency than Nec-1s in enhancing OPC proliferation in vitro.

### ZJU-37 promotes OPC proliferation within the demyelination lesion in vivo

**Fig. 4:**
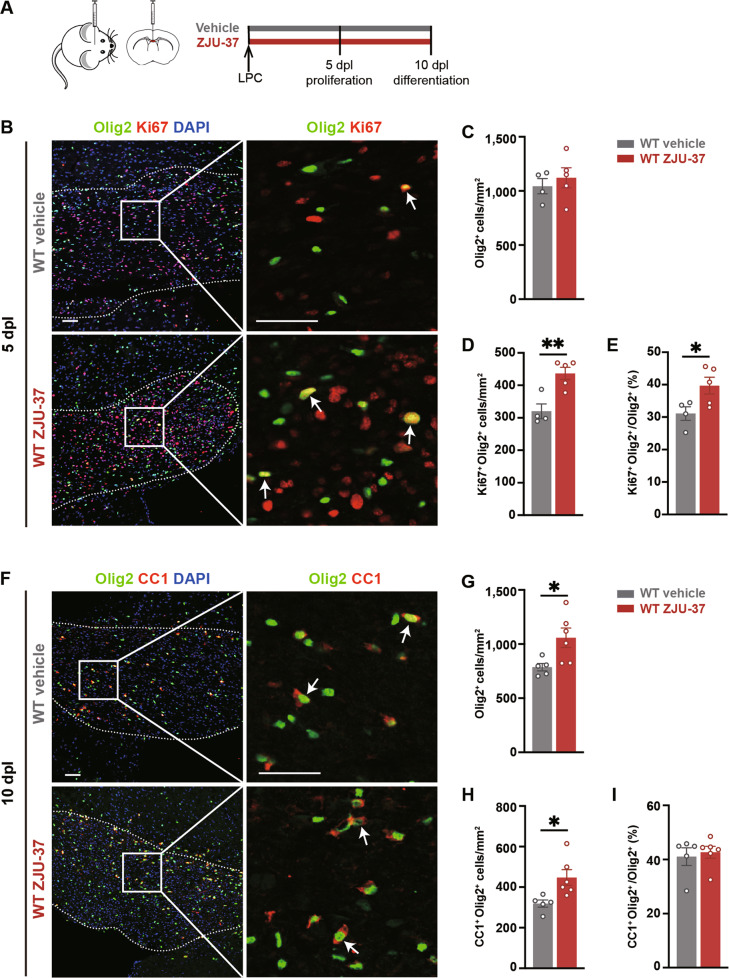
ZJU-37 promotes OPC proliferation within the demyelination lesion in vivo. **A** Schematic diagram of the experimental design. **B** Representative fluorescence images of proliferating OPCs (arrows, Olig2^+^Ki67^+^ cells) within the demyelination region (dotted line) at 5 dpl. Scale bars, 50 μm. **C**–**E** Quantification of (**C**) oligodendrocyte lineage cell numbers, (**D**) proliferating OPCs numbers and (**E**) proportion of proliferating OPCs in all oligodendrocyte lineage cells within the demyelination region at 5 dpl (two-tailed *t* test, *n* = 4 mice in the vehicle group, *n* = 5 mice in the ZJU-37 group). **F** Representative images of differentiated oligodendrocytes (arrows, Olig2^+^CC1^+^ cells) within the demyelination region at 10 dpl. DAPI (blue), Olig2 (green), CC1 (red). Scale bars, 50 μm. Quantification of (**G**) oligodendrocyte lineage cell numbers, (**H**) differentiated oligodendrocytes numbers and (**I**) proportion of differentiated oligodendrocytes in all oligodendrocyte lineage cells within the lesion region at 10 dpl (two-tailed *t* test, *n* = 5 mice in the vehicle group, *n* = 6 mice in the ZJU-37 group). All data are presented as mean ± SEM. *P < 0.05, **P < 0.01, ***P < 0.001, n*.*s. no significance.

In response to demyelination, OPCs are recruited into the lesion and expand their number by proliferation [1–3, 22]. To test whether ZJU-37 affects the OPC proliferation in vivo, we used a demyelination mouse model which provides clear temporal sequence of proliferation, differentiation of OPCs and remyelination [22, 23]. We tested the proliferation of OPCs within the lesion at 5 dpl when OPC proliferation peaks [20] (Fig. 4A), and found that ZJU-37 increased the density and proportion of proliferating OPCs (Ki67^+^Olig2^+^) while did not change the density of oligodendrocyte lineage cells (Fig. 4B–E), indicating that ZJU-37 promotes OPC proliferation in vivo.

Next we tested the differentiation of OPCs within the lesion at 10 dpl when OPC differentiation peaks [19, 23]. We showed that the density of oligodendrocyte lineage cells (olig2^+^) and mature oligodendrocytes (CC1^+^Olig2^+^) increased in the ZJU-37 group (Fig.4F–H) in proportion to the increased proliferating OPCs (Fig. 4D), while the proportion of mature oligodendrocytes (CC1^+^Olig2^+^/ Olig2^+^) did not change (Fig. 4F, I). The increase in the number of mature oligodendrocytes is likely due to the enhanced proliferation of OPC.

### ZJU-37 enhances remyelination in vivo

**Fig. 5:**
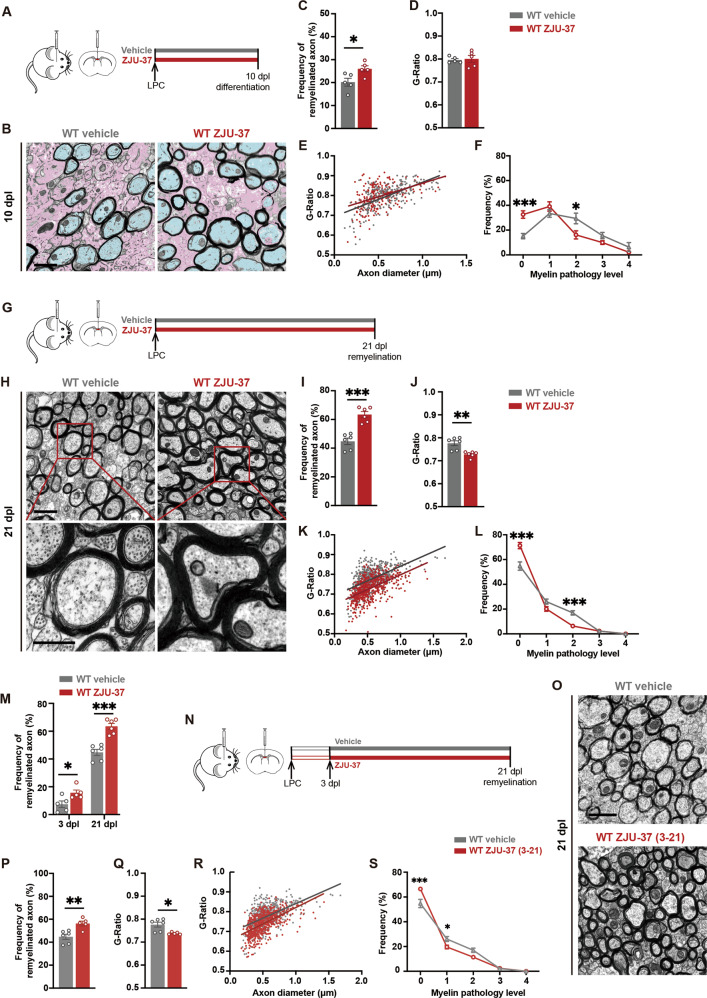
ZJU-37 enhances remyelination in vivo. **A** Schematic diagram of the experimental design. **B** Representative transmission electron micrographs within the demyelination region at 10 dpl. Scale bars, 1 μm. **C–F** Quantification of (**C**) remyelinated axons (two-tailed *t* test), (**D**) G-Ratio (two-tailed *t* test), (**E**) individual G-Ratio distribution (linear regression) and (**F**) myelin pathology level (two-way repeated ANOVA, Sidak’s post hoc test) within the demyelination region at 10 dpl (*n* = 5 mice). **G** Experimental design to test remyelination in vivo. **H** Representative electron micrographs within the demyelination region at 21 dpl. Scale bars, 1 μm (upper row), 500 nm (lower row). **I**–**L** Quantification of (**I**) remyelinated axons (two-tails *t* test), (**J**) G-Ratio (two-tailed *t* test), (**K**) individual G-Ratio distribution (linear regression) and (**L**) myelin pathology level (two-way repeated ANOVA, Sidak’s post hoc test) within the demyelination region at 21 dpl (*n* = 6 mice). **M** Quantification of remyelinated axons within the demyelination region at 3 dpl and 21 dpl (two-way repeated ANOVA, Sidak’s post hoc test). **N** Experimental design to test the effect of ZJU-37 on remyelination after completion of demyelination (ZJU-37 treatment started on the third day post LPC injection when demyelination has finished). **O** Representative transmission electron micrographs within the demyelination region at 21 dpl. **P**–**S** Quantification of (**P**) the remyelinated axons (two-tailed *t* test), (**Q**) G-Ratio (two-tailed *t* test), (**R**) individual G-Ratio distribution (linear regression) and (**S**) the myelin pathology level (two-way repeated ANOVA, Sidak’s post hoc test) within the demyelination region at 21 dpl (*n* = 5 mice). All data are presented as mean ± SEM. *P < 0.05, **P < 0.01, ***P < 0.001, n.s. no significance.

To test the effect of ZJU-37 on remyelination in vivo, we used the focal LPC-induced demyelination model, and the remyelination was evaluated under TEM. At 10 dpl (Fig. 5A) when remyelination emerges, we confirmed a significant increase in the number of remyelinated axons (light blue) in ZJU-37 treatment group whereas more axons still remained demyelinated (pink) in the vehicle group (Fig. 5B). The frequency of remyelinated axon increased to 26% of total axons after ZJU-37 treatment (Fig. 5C). Unchanged G-Ratio value reflects no difference between two groups in the thickness of newly formed myelin (Fig. 5D, E). We established a set of graded evaluation criteria on ultrastructure of myelin in the corpus callosum from grade 0 to grade 4 which is described in detail in the Methods. By this set of criteria we found that the frequency of grade 0 (normal) myelin increased while the frequency of grade 2 myelin decreased by ZJU-37 treatment (Fig. 5F, L). At 21 dpl (Fig. 5G, H) when remyelination completes, both the frequency of remyelinated axons (Fig. 5I) and the thickness of newly formed myelin (Fig. 5J, K) increased by ZJU-37. Our data indicate that ZJU-37 not only accelerates the process of remyelination, but also improves the quality of newly formed myelin.

At 3 dpl when demyelination completes whereas remyelination has not started [19, 20], the difference in the frequency of remyelinated axons between ZJU-37 and control reflects the effects of ZJU-37 on protecting myelin from demyelination (Fig. 2A–D, Fig. 5M). In comparison, at 21 dpl when remyelination completes, this difference doubled (Fig. 5M), suggesting that ZJU-37 enhancing remyelination could be caused by both protecting myelin from demyelination and directly promoting proliferation of OPCs in vivo.

To explore the potential of ZJU-37 as a treatment, the ZJU-37 or vehicle was i.p. injected starting from 3 dpl when demyelination has fully established [19, 23], and injected daily until tissue fixation (Fig. 5N). We found that at 21 dpl (Fig. 5O), both the frequency of remyelinated axons (Fig. 5P) and the thickness of newly formed myelin increased (Fig. 5Q, R). The frequency of normal-looking newly formed myelin (grade 0) increased while the grade 1 myelin decreased (Fig. 5S) by ZJU-37. These results indicate that, remarkably, ZJU-37 enhances remyelination as a treatment after demyelination established.

### ZJU-37 promotes OPC proliferation and enhances remyelination by inhibiting RIPK1 kinase activity

**Fig. 6:**
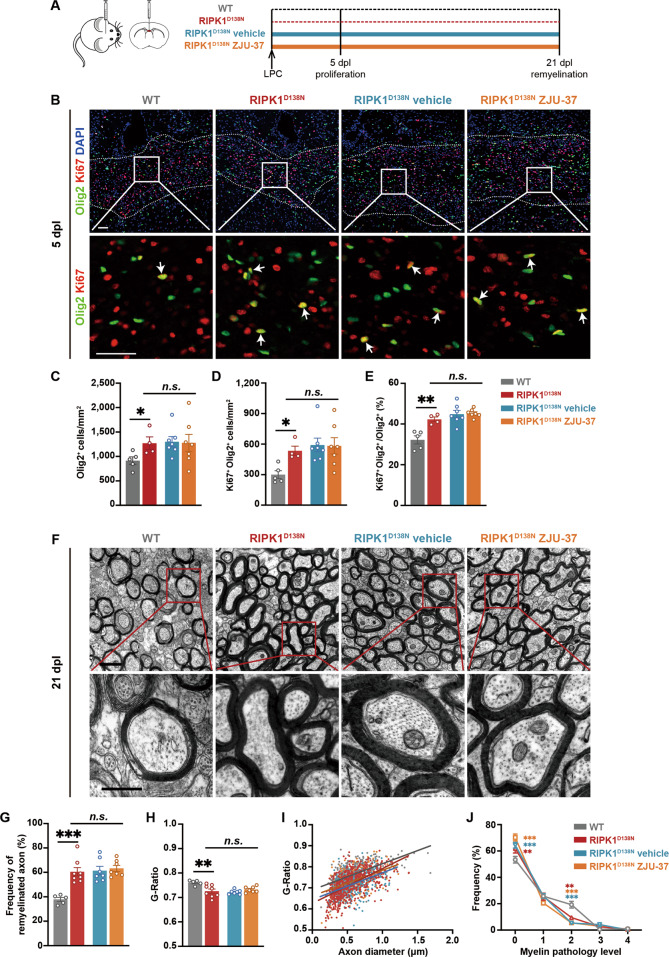
ZJU-37 promotes OPC proliferation and enhances remyelination by inhibiting RIPK1 kinase activity. **A** Schematic diagram of the experimental design. **B** Representative fluorescence images of the proliferating OPCs within the demyelination region at 5 dpl. Scale bars, 50 μm. **C**–**E** Quantification of (**C**) the oligodendrocyte lineage cell numbers, (**D**) the proliferating OPCs numbers and (**E**) the proportion of proliferating OPCs in all oligodendrocyte lineage cells within the lesion region at 5 dpl (one-way ANOVA, Tukey’s post hoc test, *n* = 5 mice in the WT group, *n* = 4 mice in the RIPK1^D138N^ group, *n* = 7 mice in the RIPK1^D138N^ vehicle and RIPK1^D138N^ ZJU-37 group). **F** Representative transmission electron micrographs within the demyelination region at 21 dpl. Scale bars, 1 μm (upper row), 500 nm (lower row). **G**–**J** Quantification of (**G**) the remyelinated axons (one-way ANOVA, Tukey’s post hoc test), (**H**) G-Ratio (one-way ANOVA, Tukey’s post hoc test), (**I**) individual G-Ratio distribution (linear regression) and (**J**) the myelin pathology level (two-way repeated ANOVA, Sidak’s post hoc test) within the demyelination region at 21 dpl (*n* = 5 mice per group). All data are presented as mean ± SEM. *P < 0.05, **P < 0.01, ***P < 0.001, n.s. no significance.

It has been shown that the RIPK1 D138N kinase-dead knock-in mutation is deficient both to be inhibited by RIPK1 inhibitors and in TNFα-induced necroptosis [24, 25]. We cultured OPCs from the RIPK1^D138N^ and the WT mice and found that the proliferation of the RIPK1^D138N^ OPCs was much higher than that of the WT OPCs, and ZJU-37 treatment did not change the proliferation of the RIPK1^D138N^ OPCs (Supplementary Fig. 2A, B), confirming that ZJU-37 promotes OPC proliferation by inhibiting RIPK1 kinase activity. To ask whether the effect of ZJU-37 on remyelination is mediated by inhibition of RIPK1 kinase activity in vivo, we induced demyelination in the RIPK1^D138N^ and the WT mice and i.p. injected ZJU-37 or vehicle into the RIPK1^D138N^ mice daily until tissue fixation (Fig. 6A). The results showed that at 5 dpl, the density of oligodendrocyte lineage cells, the density and the proportion of proliferating OPCs consistently increased within the lesion of the RIPK1^D138N^ mice compared to the WT mice (Fig. 6B–E). Importantly, none of the three indexes was affected by ZJU-37 in the RIPK1^D138N^ mice (Fig. 6B–E). Collectively, these results confirmed that ZJU-37 promotes OPC proliferation by inhibiting RIPK1 kinase activity.

At 21 dpl, under TEM, we showed that the frequency of remyelinated axon significantly increased in the RIPK1^D138N^ mice (Fig. 6F, G), and the newly formed myelin in the RIPK1^D138N^ mice was thicker than the WT mice (Fig. 6H, I). The frequency of normal-looking newly formed myelin (grade 0) increased in the RIPK1^D138N^ mice while the grade 2 myelin decreased, indicating that the quality of the newly formed myelin in the RIPK1^D138N^ mice was much better than the WT mice (Fig. 6J). Remarkably, neither of these indexes was affected by ZJU-37 in the RIPK1^D138N^ mice (Fig. 6F–J), demonstrating that ZJU-37 promotes remyelination by inhibiting RIPK1 kinase activity in vivo.

### ZJU-37 promotes transcription of PDGFRα and proliferation of OPCs via NF-κB

**Fig. 7:**
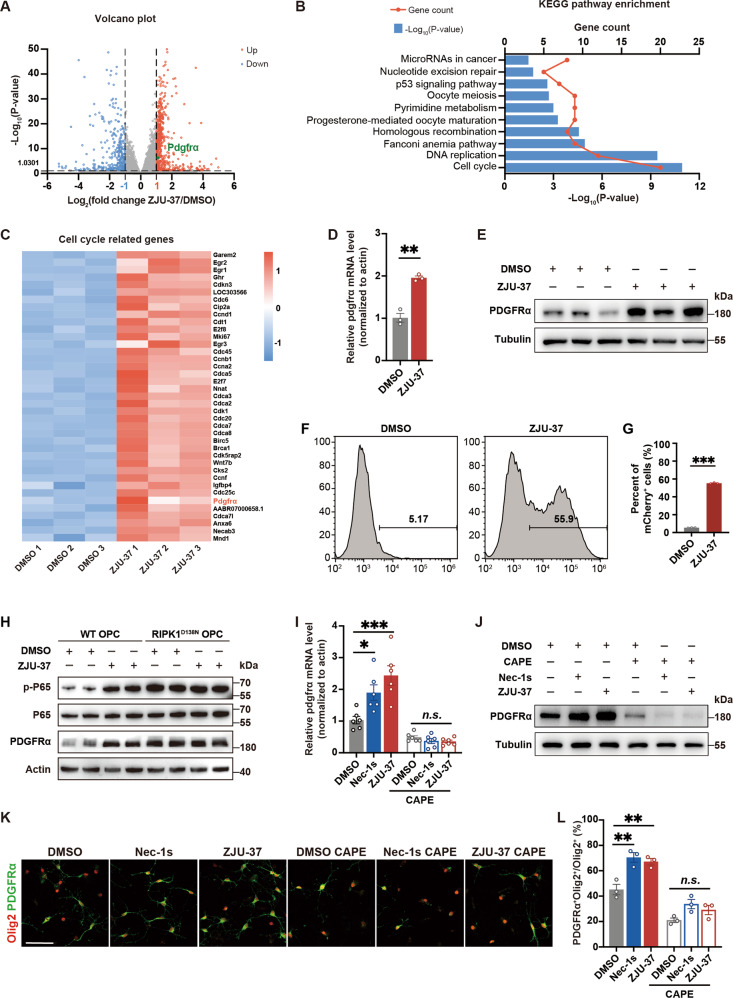
ZJU-37 promotes transcription of PDGFRα and proliferation of OPCs via NF-κB. **A** Volcano plot of 2575 genes that are differentially regulated by ZJU-37 or DMSO in primary rat OPCs treated for 36 h (*n* = 3 plate wells). **B** KEGG pathway enrichment analysis of genes upregulated by ZJU-37 in OPCs. **C** Heat map of the cell cycle related genes in rat OPCs under indicated treatments. High expression is shown in red and low expression in blue. **D** The mRNA level of PDGFRα in rat OPCs was verified by qPCR (two-tailed *t* tests, *n* = 3 plate wells). **E** The protein level of PDGFRα in rat OPCs was measured by immunoblotting. **F** Flow cytometry chart of Jurkat cells treated with ZJU-37 or DMSO. **G** Quantification of mCherry^+^ Jurkat cells treated with ZJU-37 or DMSO (two-tailed *t* test, *n* = 3 plate wells). **H** The protein levels of p-P65 and PDGFRα in WT and RIPK1^D138N^ OPCs were measured by immunoblotting. **I** The mRNA level of PDGFRα in rat OPCs under various treatments was detected by qPCR (one-way ANOVA, Tukey’s post hoc test, *n* = 3 plate wells). CAPE, inhibitor of NF-κB, 2 μM. **J** The protein level of PDGFRα in rat OPCs under various treatments was measured by immunoblotting. **K** Representative images of proliferating OPCs from primary rat after 36 h treatment of DMSO, Nec-1s, ZJU-37 or ± CAPE. Proliferating OPCs was visualized by Olig2 (red) and PDGFRα (green). Scale bars, 50 μm. **L** Quantification of the OPC proportion under virous treatments (one-way ANOVA, Tukey’s post hoc test, *n* = 3 plate wells). All data are presented as mean ± SEM. *P < 0.05, **P < 0.01, ***P < 0.001, n.s. no significance.

To explore how ZJU-37 regulates the transcriptional network in OPCs, we analyzed the ZJU-37 regulated gene transcription of OPCs isolated from the brains of new-born rat by RNA sequencing (RNA-seq). The volcano plot analysis showed a total of 2575 genes were detected and 518 genes were upregulated whereas 322 genes were downregulated by ZJU-37 (Fig. 7A). The KEGG pathway analysis showed that the most upregulated pathway was the cell cycle pathway (Fig. 7B). Interestingly, a well-known OPC proliferation marker PDGFRα was among the 39 upregulated cell cycle related genes induced by ZJU-37 (Fig. 7C), and the upregulation of PDGFRα was verified at level of both mRNA (Fig. 7D) and protein (Fig. 7E). When OPCs were treated with BLU-285, a specific inhibitor of PDGFRα, we found that BLU-285 completely blocked ZJU-37-mediated promotion of OPC proliferation (Supplementary Fig. 3A, B), indicating that PDGFRα is critical for the effect of ZJU-37 on promoting OPC proliferation.

Given that transcription factor NF-κB can be regulated by RIPK1 [17], we used the Jurkat-NF-κB-mCherry reporter cell line in which the activation of NF-κB is reflected by the mCherry fluorescence. Our flow cytometry data showed that ZJU-37 sharply increased the proportion of NF-κB-activated Jurkat cells from 5.17% to 55.9% (Fig. 7F, G), confirming that ZJU-37 directly activates NF-κB in Jurkat cells. We further detected the phosphorylation level of P65 in primary cultured OPCs from WT and RIPK1^D138N^ mice which has a RIPK1 kinase dead mutation. The level of p-P65 was enhanced by ZJU-37 in WT OPCs (Fig. 7H), however, in OPCs of RIPK1^D138N^ mice, the level of p-P65 was inherently high and was not affected by ZJU-37 (Fig. 7H). These results verified that ZJU-37 activates NF-κB in OPCs by inhibiting RIPK1 kinase activity indeed.

Since PDGFRα is essential for OPC proliferation [26, 27], and ZJU-37 promotes OPC proliferation and activates NF-κB, we wonder whether NF-κB involves in ZJU-37 upregulating PDGFRα. We showed that ZJU-37 upregulated PDGFRα in OPCs of WT mice but did not affect PDGFRα in OPCs of RIPK1^D138N^ mice (Fig. 7H). By using caffeic acid phenethyl ester (CAPE), an inhibitor of NF-κB, we found that inhibition of NF-κB abolished the effects of both ZJU-37 and Nec-1s on the transcription of PDGFRα to the control level (Fig. 7I, J). Both pharmacological and genetic evidence consistently confirmed that NF-κB is critical for the effects of ZJU-37 on promoting transcription of PDGFRα. Furthermore, we found that CAPE deleted the effect of ZJU-37 and Nec-1s on promoting OPC proliferation (Fig. 7K, L), indicating that NF-κB is critical for both ZJU-37 and Nec-1s to promote proliferation of OPCs. Together, these results revealed that ZJU-37, a novel inhibitor of RIPK1, promotes transcription of PDGFRα and proliferation of OPCs via NF-κB.

## Discussion

We identified here that ZJU-37 exhibits higher potency on TNFα induced necroptosis than Nec-1s, the most potent inhibitor of RIPK1 activity reported by far [10, 12]. Compared with its original form Nec-1s, ZJU-37 introduces an amide bond modification which makes ZJU-37 more potent than Nec-1s. RIPK1, a key mediator of necroptosis, controls inflammation and cell death via its kinase domain [28], and mediates the multimodal signaling pathways downstream to TNFR1 [29]. Since RIPK1 activation and necroptosis have been genetically and mechanistically linked with human neurodegenerative diseases such as MS [8, 9, 14], ALS [18] and Alzheimer’s disease (AD) [30, 31], keen interests have been sparkled on developing CNS-penetrant new RIPK1 inhibitors as drug candidates to treat CNS diseases [17]. RIPK1 inhibitors, such as GSK2982772 [32] and DNL747 [33], have successfully completed their phase II clinical trials [12], warranting the safety and displaying their effectiveness in human subjects. Here we screened out a series of novel RIPK1 inhibitors including several drug candidates with previously unknown targets, and ZJU-37 exhibits the highest potency on TNFα induced necroptosis in vitro. We further showed that ZJU-37 protects myelin from demyelination and axons from degeneration in LPC-induced demyelination in vivo. The dual protective effects of ZJU-37 are consistent with previous reports using other RIPK1 inhibitors [18, 21, 34].

Remarkably, here we have established a new role of RIPK1 activity inhibitor on pro-remyelination by enhancing the proliferation of OPCs through inhibiting RIPK1 activity during which ZJU-37 exhibits higher potency than Nec-1s and therapeutic effects. Demyelination is a common key pathology not only for MS [1, 2] but also for neurodegenerative diseases such as AD, Parkinson’s disease [35], ALS [18], and Huntington’s disease [36], and demyelination can be healed through remyelination which declines with progressive aging partially due to the depletion of OPCs [5, 6, 22, 37]. To achieve effective remyelination, it has long been focused on enhancing differentiation of OPCs [2, 3], and different drugs such as Benztropine, Olesoxime and Vitamin C and several ongoing clinical trials aim to enhance the differentiation of OPCs in MS [4, 7, 38–40]. However, to our knowledge, no drug or ongoing clinical trial can enhance proliferation of OPCs [4, 7]. Our study unveils a previously unexplored function of RIPK1 in enhancing the proliferation of OPCs and promoting remyelination by inhibiting RIPK1 kinase activity, and reveals ZJU-37 is a promising new drug candidate for demyelinating diseases, suggesting that promoting proliferation of OPCs is another promising strategy to enhance remyelination.

Mechanistically, we revealed that ZJU-37 promotes proliferation of OPCs by enhancing transcription of PDGFRα which is essential for OPCs proliferation [26, 27], and its agonist PDGFAA is a major in vivo mitogen for the OPCs especially the adult OPCs [41, 42]. Interestingly, NF-κB is critical for ZJU-37 on upregulating PDGFRα and promoting proliferation of OPCs. NF-κB is a key regulator downstream to RIPK1 which is expressed in OPCs in this and previous studies [43] and is involved in regulating inflammation [44], cell survival [45, 46], and myelination of the peripheral nerve [47]. These data suggest that its possible mechanisms involve RIPK1-NF-κB-PDGFRα pathway. The detailed mechanisms underlying the effects of ZJU-37 is intriguing and deserves further studies. Taking previous reports into account, it is reasonable to propose that ZJU-37 exhibits pro-remyelination effects probably by targeting multiple cell types such as OPCs, microglia [14, 23, 48] and astrocytes [14] through multiple mechanisms.

In conclusion, we identify ZJU-37, a small-molecule compound modified from Nec-1s, as a new RIPK1 kinase inhibitor with higher potency than Nec-1s, and uncovers in addition to its protective effects on both myelin and axons, ZJU-37 exhibits new role on promoting proliferation of OPCs and enhancing remyelination by inhibiting RIPK1 kinase activity and enhancing the transcription of PDGFRα via NF-κB pathway. This work identifies ZJU-37 as a new drug candidate which enhances remyelination by promoting proliferation of OPCs, paving the way for further exploring a potential therapy for demyelinating diseases.

## Methods

### Antibodies

The antibodies used were as follows: rat anti-Ki67 (#14-5698-82, Thermo Fisher Scientific, 1:500), rabbit anti-Olig2 (#AB9610, Millipore, 1:400), rat anti-MBP (#MCA409S, Bio-Rad Laboratories, 1:500), mouse anti-CC1 (#ab16794, Abcam, 1:200), goat anti-PDGFRα (#AF1062, R&D Systems, 1:100), rabbit anti-PDGFRα (#3164 S, Cell Signaling Technology, 1:1000), mouse anti-P65 (#6956 S, Cell Signaling Technology, 1:1000), mouse anti-p-P65 (#3036 S, Cell Signaling Technology, 1:1000), mouse anti-Actin (#M1210-2, HUABIO, 1:5000), mouse anti-Tubulin (#M1305-2, HUABIO, 1:5000), mouse anti-RIPK1 (#610458, BD, 1:1000), rabbit anti-p-RIPK1 (#65746, Cell Signaling Technology, 1:1000). Secondary antibodies: Cy^TM^3 affinipure donkey anti-rat IgG (H + L) (#712-165-153, 1:400), Cy^TM^3 affinipure donkey anti-mouse IgG (H + L) (#715-165-151, 1:400), Alexa Fluor 488 affinipure donkey anti-rabbit IgG (H + L) (#711-545-152, 1:400) and Alexa Fluor 488 affinipure donkey anti-goat IgG (H + L) (#705-545-003, 1:400) were purchased from Jackson ImmunoResearch; Goat anti-mouse IgG (H + L) conjugated with HRP (#31430, 1:20000), goat anti-rabbit IgG (H + L) conjugated with HRP (#31,460, 1:20000) were purchased from Thermo Fisher Scientific.

### Cell lines and cell culture

All cell lines were cultured at 37 °C with 5% CO_2_. Fas-associated via death domain (FADD)-deficient human Jurkat T cells (Jurkat FADD^−/−^), human tissue cell lymphoma cell line (U937) and human embryonic kidney cell line (HEK293T) were gifts from Dr. Junying Yuan (Harvard medical school, Boston). Human cell lines were cultured as follows: BV2 and HEK293T in DMEM (#SH30022.01, HyClone) with 10% FBS (#3682, Internegocios-sa) and 1% Penicillin /Streptomycin (#15140–122, GIBCO); Jurkat FADD^−/−^ and U937 in RPMI-1640 (#SH30809.01, HyClone) with 10% FBS and 1% Penicillin/Streptomycin. Lipofectamine™ 2000 (#11668–019, Invitrogen™) was used for transfection of HEK293T cells.

### Animals

C57BL/6, RIPK1^D138N^ mice were used in this study. C57BL/6 mice were purchased from Model Animal Research Center of Nanjing University (Nanjing, Jiangsu, China). RIPK1^D138N^ mice were from the Pengyu Huang Laboratory and verified by tail DNA polymerase chain reaction (PCR) using primers (5′-ttccctttcctcgtaacccc-3′ and 5′-ggcctcagaatcctccactt -3′).

To test the effect of ZJU-37 in vivo, ZJU-37 (10 mg/kg bodyweight) or vehicle (2% DMSO + 30% PEG400 + 68% ddH_2_O) were intraperitoneally (i.p.) injected into the mice half an hour before the LPC injection and then injected ZJU-37 or vehicle daily until tissue fixation. All experimental mice were group-housed in a light-dark (LD) 12:12 cycle temperature-controlled room and given ad libitum access to food and water in Zhejiang University Animal experimental center. Male mice were at least 6–8 weeks old at the time of experiment starting. Littermates were used for studies comparing mutant and WT mice. The mice were randomly assigned to the experimental group and the control group. The experimenters were blinded to the grouping of the mouse until the data were integrated. All experiments with mice were performed with the ethical approval of the Zhejiang University Animal Care Committee.

### High-throughput screening

FDA-approved drug or drug candidate library was purchased from Topscience, Inc. (Shanghai, China). 8 × 10^3^ Jurkat FADD^−/−^ cells were treated with TNFα and indicated compounds for 12 h and seeded in 384-well plates per well. Treatments were performed with three repeats; Each plate includes negative control (DMSO) and positive control (Nec-1s). The screen Z-factor was > 0.5. Cell viability was estimated by ratio of live cells and measured using CellTiter-Glo luminescent cell viability assay (#G7573, Promega). High-throughput was done using Biotek Cytation^®^ 3.

### Western blot analysis

For western blot analysis, cells were lysed in RIPA buffer (0.5% NP-40, 150 mM NaCl, 1 mM NaF, 1 mM EDTA, 1 mM Na_3_VO_4_ and 50 mM Tris-HCl-pH7.5, protease inhibitors, phosphatase inhibitors) and incubation on ice for 30 min. The lysate was centrifuged at 12,000 rpm, 4 °C for 10 min. Then the supernatant was collected and the protein concentration was quantified by bicinchoninic acid (BCA) reaction (#160921E, Biotech). 2× SDS-PAGE loading buffer was added in samples and heated at 100 °C for 10 min. Samples were separated by SDS-PAGE gels and transferred to PVDF membranes. Blocking was performed in 5% nonfat dried milk (Wandashan) or BSA (9048-46-8, Sangon Biotech) diluted in PBS-T (PBS, 1% Tween-20). Incubation of all primary antibodies was performed on shaker at 4 °C overnight. Incubation of all secondary antibodies was performed on shaker at room temperature for 1 h. All washing steps were performed using PBS-T (10 min, 3 times). Detection was performed using HRP-conjugated secondary antibodies and chemiluminescence reagents (#4 AW001-500, Beijing 4A Biotech Co., Ltd.).

### Scanning electron microscope

After Jurkat FADD^−/−^ cells were treated with indicated compounds, cells were fixed on slides with 2.5% glutaraldehyde solution at room temperature for 1–2 h and transferred to 4 °C for overnight fixation; The samples were then washed with freshly prepared 0.1 M, pH 7.4 phosphoric acid buffer (PBS) (15 min, 3 times) and were post-fixed in 1% osmium-tetroxide for 1.5 h. Next, the samples were washed with freshly prepared 0.1 M, pH 7.4 PBS (15 min, 3 times) and dehydrated in 30%, 50%, 70%, 80% and 90% acetone each for 15 min and 100% acetone for 20 min (2 times). Finally, the samples were dried at critical point and observed by SEM (Nova Nano 450, Thermo FEI).

### EC_50_ determination of cell viability

The EC_50_ was determined in FADD-deficient Jurkat cells treated with human TNFα (#300–01 A, Peprotech). FADD-deficient Jurkat cells were cultured in 96-well white plate and treated with indicated compounds. The cell viability was estimated by ratio of live cells and was measured using CellTiter-Glo luminescent cell viability assay (#G7573, Promega). EC_50_ was calculated (viability, %) by nonlinear regression in GraphPad Prism.

### In vitro kinase assay

HEK293T cells were transfected with Flag-RIPK1 for 48 h, Flag-RIPK1 was immunoprecipitated from HEK293T cells using anti-FLAG agarose beads (#B23102, Bimake) for 6 h on a rotating wheel at 4 °C. Next washed with lysis buffer (5% glycerol, 500 mM NaCl, 0.5% NP-40 and 50 mM Tris-HCl (pH7.4), protease inhibitors, phosphatase inhibitors) for 3 times and then washed with water for 2 additional times. Flag agarose beads were resuspended in 30 μL reaction buffer (20 mM MgCl_2_, 2 mM DTT, 40 mM Tris-HCl (pH7.5) and 50 μM inhibitors as indicated) after the final wash step. Kinase reactions were performed for 30 min at 30 °C (mix it every 5–10 min) and arrested by the addition of 20 μL 2× loading buffer and heated at 100 °C for 10 min. RIPK1 kinase activity was evaluated by Western blot with anti-p-RIPK1 Ser166 antibody.

### Primary cell culture

For rat OPC culture, the cerebral cortices of 2 SD rats at postnatal day 0–2 were dissected out in ice-cold HBSS (#14025092, Gibco). After digestion in 0.25% trypsin (#25200056, Gibco) for 12 min at 37 °C, the tissue was blown into a single cell suspension in DMEM/F12 (#11320082, Gibco) containing 10% fetal bovine serum (FBS, #10099141, Gibco) and then centrifuged at 1400 rpm for 5 min at 4 °C. The cells were resuspended and plated in a 75 cm^2^ tissue culture flask pre-coated with poly-D-lysine (PDL, #P0899, Sigma, 0.1 mg/ml). Cells were incubated at 37 °C, 5% CO_2_ and maintained for about 7 days with a medium change every 3 days. For cell purification, the flask was placed on the shaker at 200 rpm for 2 h at 37 °C to remove microglia cells and then was shaken at 250 rpm for 16–18 h after medium was changed. Then replaced OPCs onto coverslips coated with PDL in 24 well tissue culture dishes. After 3–4 h, the culture medium was changed to Neurobasal (#21103,049, Gibco) containing B27 (#17504,044, Gibco) and N2 (#17502,048, Gibco). The next experiment can be performed the next day.

For mouse OPC culture, the cerebral cortices of 4 mice at postnatal day 0–2 were dissected out in ice-cold HBSS. Then the tissue was minced and gently blown into cell suspension with a pipette. The cell suspension was transferred into a 75 cm^2^ tissue culture flask pre-coated with PDL and maintained in DMEM/F12 containing 20% FBS and incubated at 37 °C, 5% CO_2_. The medium was completely changed after 3 days and the cells were maintained in the same medium for about 7 days with a medium change every 2 days. The following steps are the same as for the rat OPC.

### Immunofluorescence labelling for cells

Cultured cells were rinsed with PBS 3 times and fixed with 4% PFA for 10 min. Then cells were washed 3 times with PBS and blocked with 5% normal donkey serum (NDS, #D9663, Sigma), 0.3% Triton X-100 (#T8787, Sigma) in PBS for 2 h at 4 °C. The cells were incubated with primary antibodies (diluted with 2.5% NDS, 0.3% Triton X-100 in PBS) for 4 h at room temperature and secondary antibodies (diluted with 2.5% NDS, 0.3% Triton X-100 in PBS) for 2 h at 4 °C. For nuclei staining, the cells were incubated with DAPI (#D9542, sigma,0.1 μg/ml) for 10 min at room temperature and were washed 3 times with PBS. Then the coverslips were mounted onto glass slide in a drop of Flurosave^TM^ Reagent (#345,789, Millipore). Images were acquired using Olympus FV1200 microscope and further image processing and analysis was performed using the ImageJ software.

### Immunofluorescence labelling for tissue sections

Mice were anesthetized with sodium pentobarbital (0.1 g/kg) by i.p. injection and transcardially perfused with 4% paraformaldehyde (PFA) in 0.1 M PB (pH 7.4). The brain was post-fixed for 2 h at 4 °C and immersed into up-graded sucrose: 10% and 20% for 2 h, 30% in 0.1 M PB until it sank to the bottom. The brain was then embedded in tissue freezing medium (#03813266, Leica) and sliced into sections at thickness of 12 μm using a cryostat (Leica CM 1950). The slides were washed with PBS (5 min, 3 times) to wash away the tissue freezing medium and then placed into a pre-heated citrate buffer (pH 6.0) in a water bath for 15 min for antigen-retrieval. After washing with PBS (5 min, 3 times), the slides were blocked with 5% NDS, 0.3% Triton X-100 in PBS for 2 h at 4 °C. The slides were incubated with primary antibodies (diluted with 2.5% NDS, 0.3% Triton X-100 in PBS) for about 48 h at 4 °C and then secondary antibodies (diluted with 2.5% NDS, 0.3% Triton X-100 in PBS) for 2 h at 4 °C. The slides were incubated with DAPI for 10 min at room temperature and finally washed with PBS (5 min, 3 times). Then the slides were mounted with coverslips using FluroSave. Images were acquired using Olympus FV1200 microscope and further image processing and analysis was performed using the ImageJ software.

### White matter demyelination induction

Mice were anesthetized with i.p. injection of sodium pentobarbital (0.1 g/kg). 2 μL 1% lysolecithin (LPC, #L4129, Sigma) was injected into the middle of corpus callosum (bregma 0.5 mm) to induce demyelination. LPC was delivered at a rate of 0.2 μL/min. The needle was pulled out slowly after remained in position for 10 min and then sutured the wound. The mice were placed on a heating pad until woke up.

### Transmission electron microscope

Mice were anesthetized with sodium pentobarbital (0.1 g/kg) by i.p. injection and transcardially perfused with 4% glutaraldehyde in 0.1 M PB (pH 7.4). The lesion region of corpus callosum (1 mm^3^) were dissected and immersed into 4% glutaraldehyde at 4 °C for at least 24 h. The samples were washed with 0.1 M cacodylate buffer (10 min, 3 times) and fixed in 2% osmium-tetroxide for 1 h on ice. Then the samples were washed with deionized water (5 min, 4 times) and incubated for 1 h on ice in 4% aqueous uranyl acetate, and then washed with deionized water (5 min, 4 times). Next, the samples were dehydrated in 50%, 70%, 90% and 95% acetone for 15 min and 100% acetone for 30 min (3 times). The samples were infiltrated in grades of acetate: Epon embedding mixture (1:3, 1:1, 3:1, each for 2 h) and then embedded in pure Epon mixture overnight and polymerized. The samples were sliced into 60 nm ultrathin sections and double stained with utanyl acetate and lead citrate. Images were acquired using a Tecnai G2 Spirit 120 kV transmission electron microscope and further image processing and analysis was performed using the ImageJ software.

### Assessment of remyelination

G-Ratio was measured on transmission electron micrographs using Image J. The myelin sheath can be regarded as a torus, and the areas of inner and outer circles of myelin sheath were measured with freehand tool on Image J by tracing the outer surface of each structure, then converted the areas into hypothetical diameters. The G-Ratio was calculated as the ratio of the diameter of inner circle over that of outer circle on the same myelin, which is inversely correlated to myelin thickness. The diameter of axons was calculated by area, which was also measured by tracking the outer surface of axons. The graded evaluation criteria of myelin in corpus callosum: 0, normal myelin; 1, focal split of myelin sheaths; 2, wave-like lamellae decompaction or enlarged space between lamellae; 3, myelin inclusion or redundant myelin; 4, myelin ballooned, broken or (almost) disappeared.

### RNA isolation and qRT-PCR

RNA was extracted from cultured rat OPCs using FastPure Cell/Tissue Total RNA Isolation Kit (#RC101, Vazyme). All RNA samples were stored at −80 °C until the next experiment. cDNA was generated using HiScript III 1st Strand cDNA Synthesis Kit (#R312, Vazyme). For RT-PCR, cDNA, primers and ChamQ Universal SYBR qPCR Master Mix (#Q711, Vazyme) were mixed as instructed by the manufacturer. Fold changes in gene expression were calculated using the delta delta Ct method in Microsoft Excel. Primer sequences: Pdgfrα-f, 5′-ctggctgaaggacaacttgac-3′ and Pdgfrα-r, 5′-agaatggaggcaggaactagag-3′. β-actin-f, 5′-accacagctgagagggaaatc-3′ and β-actin-f, 5′-ggtctttacggatgtcaacg-3′.

### RNA sequencing

RNA from rat OPCs treatmented with DMSO or ZJU-37 for 30 h was prepared for RNA-seq (3 biological repeats for each group). RNA-seq experiments were performed by Novogene. Total RNA was extracted from OPCs using TRIzol^TM^ Reagent (#15596026, Invitrogen^TM^). Then mRNA was purified from total RNA using poly-T oligo-attached magnetic beads. Sequencing libraries were generated using NEBNext^®^ UltraTM RNA Library Prep Kit for Illlumina^®^ (#E7770, NEB) and index codes were added to attribute sequences to each sample. The index-coded samples clustering was performed on a cBot Cluster Generation System using TruSeq PE Cluster Kit v3-cBot-HS (Illumia) according to the manufacturer’s instruction. Then the library preparations were sequenced on an Illumina Hiseq platform and 125 bp/150 bp paired-end reads were generated.

For data analysis, raw data (raw reads) of fastq format were firstly processed through in-house perl scripts. Reference genome and gene model annotation files were downloaded from genome website directly. FeatureCounts v1.5.0-p3 was used to count the reads numbers mapped to each gene. Differential expression analysis of two groups was performed using the DESeq2 R package (1.16.1). The resulting *p*-values were adjusted using the Benjamini and Hochberg’s approach for controlling the false discovery rate. The differential gene expression heat map was generated with the ggplot library.

### Generation of Jurkat-NF-κB-mCherry cell line

The NF-κB reporter cassette containing a response element for NF-κB (ggggactttccgc), a minimal promoter and a mCherry fluorescence reporter gene was inserted into a neomycin-resistant retroviral vector. Jurkat (E6-1) cells were transduced with retrovirus produced from 293 T cell lines. The cells expressing the NF-κB reporter cassette were enriched by G418 selection. A cell clone with the lowest reporter gene expression in resting state and the highest fluorescence level upon PMA/Ionomycin stimulation was selected for further usage.

### Statistical analysis

All data analysis was carried out using GraphPad Prism 8.0. Unpaired *t* test was used for comparison between two groups. One-way ANOVA followed by Tukey’s post hoc test or two-way ANOVA followed by Sidak’s multiple comparisons test were used for multiple comparisons. Data are shown as mean ± SEM. Differences were considered statistically significant at *p < 0.05, **p < 0.01 or ***p < 0.001.

## Supplementary information

Response letter to Editorial requests

Supplementary Figure 1.

Supplementary Figure 2.

Supplementary Figure 3.

Supplementary figure legends.

Supplementary Table 1.

Supplementary Table 2.

## Data Availability

The transcriptome data sets are available at NCBI (GEO accession: GSE172497).
