# Supplementary figures and images for: Inhibition of RIPK1 by ZJU-37 promotes oligodendrocyte progenitor proliferation and remyelination via NF-κB pathway
Source: Cell Death Discov. 2022 Apr 1;8:147. doi: 10.1038/s41420-022-00929-2 (PMC8975999; doi:10.1038/s41420-022-00929-2)

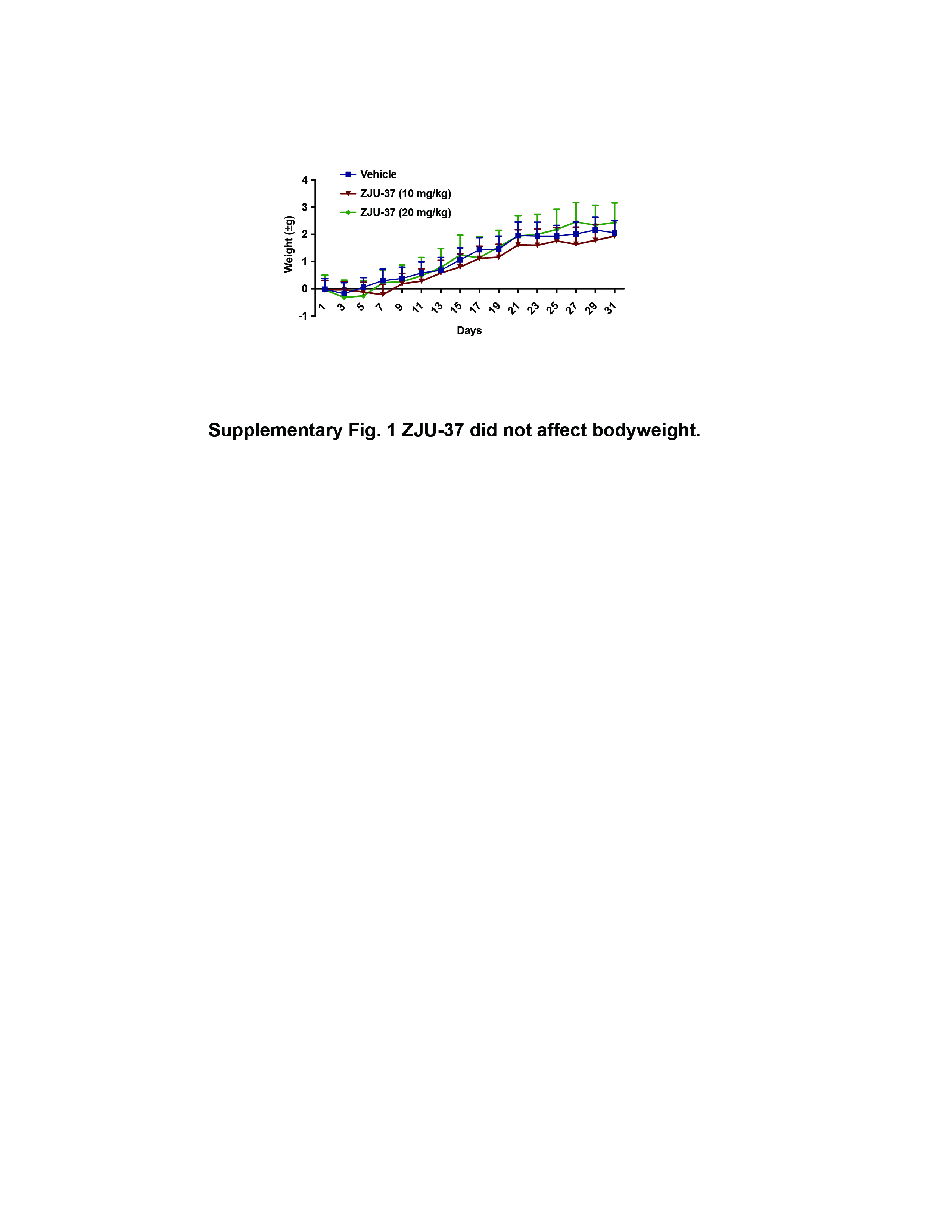

Supplement: Supplementary file 2 — Supplementary Figure 1. [file 41420_2022_929_MOESM2_ESM.tif]

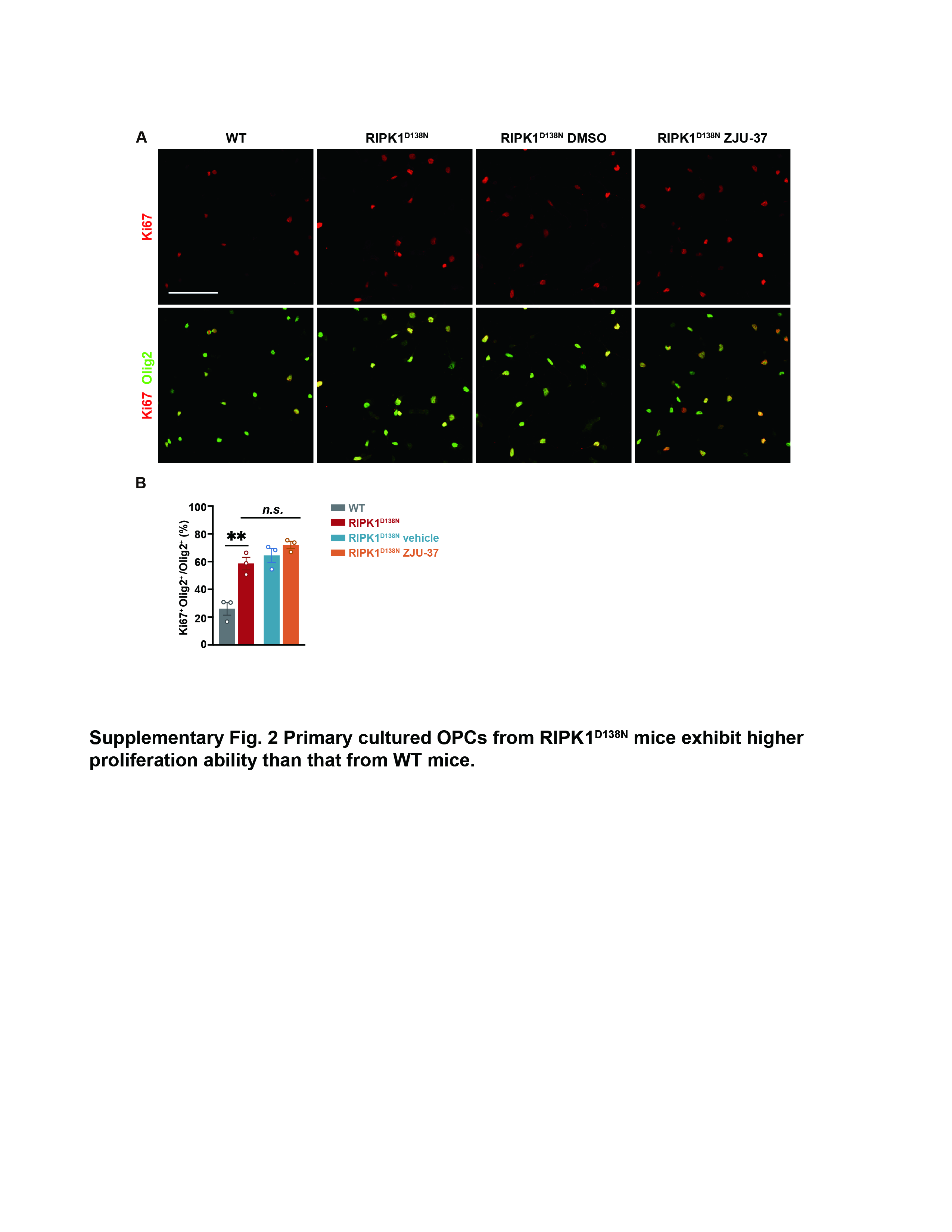

Supplement: Supplementary file 3 — Supplementary Figure 2. [file 41420_2022_929_MOESM3_ESM.tif]

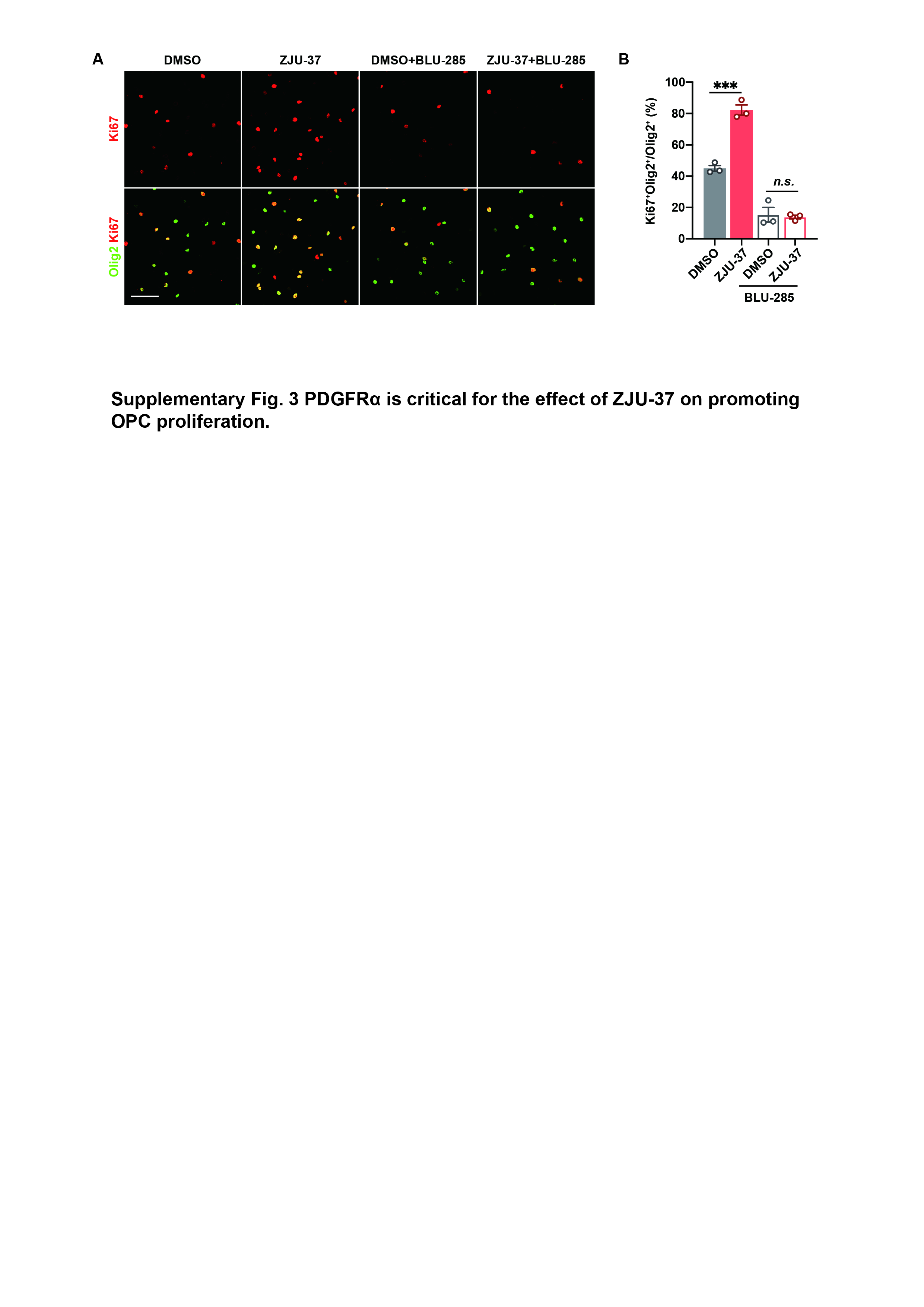

Supplement: Supplementary file 4 — Supplementary Figure 3. [file 41420_2022_929_MOESM4_ESM.tif]
