# Supplementary material for: Inhibition of RIPK1 by ZJU-37 promotes oligodendrocyte progenitor proliferation and remyelination via NF-κB pathway
Source: Cell Death Discov. 2022 Apr 1;8:147. doi: 10.1038/s41420-022-00929-2 (PMC8975999; doi:10.1038/s41420-022-00929-2)
Supplement: Supplementary file 5 — Supplementary figure legends. [file 41420_2022_929_MOESM5_ESM.docx]

**Supplementary figure legends**

**Supplementary Fig. 1 ZJU-37 did not affect bodyweight.** Bodyweight of WT mice receiving ZJU-37 or vehicle injection intraperitoneally daily. All data are presented as mean ± SEM.

**Supplementary Fig. 2 Primary cultured OPCs from RIPK1^D138N^ mice exhibit higher proliferation ability than that from WT mice.** **A** Representative fluorescence image of primary cultured OPCs from WT and RIPK1^D138N^ mice. Proliferating OPCs was visualized by Ki67 (red) and Olig2 (green). Scale bars, 50 μm. **B** Quantification of the proportion of proliferating OPCs (two-tailed *t* test, *n*=3 plate cells). All data are presented as mean ± SEM. **P* < 0.05; ***P* < 0.01; ****P* < 0.001; *n.s.*: no significance.

**Supplementary Fig. 3 PDGFRα is critical for the effect of ZJU-37 on promoting OPC proliferation. A** Representative fluorescence images of proliferating primary rat OPCs treated with ZJU-37 (5 μM) ± BLU-285 (0.5 μM) or DMSO ± BLU-285 (0.5 μM) for 36 hours. BLU-285, avapritinib (#S8553, Selleck), inhibitor of PDGFRα. Proliferating OPCs was visualized by Ki67 (red) and Olig2 (green). Scale bar: 100 μm. **B** Quantification of proliferating OPCs (one-way repeated ANOVA, Tukey’s post hoc test, *n*=3 plate wells). All data are presented as mean ± SEM. **P* < 0.05; ***P* < 0.01; ****P* < 0.001; *n.s.*: no significance.
