# Supplementary material for: Inhibition of RIPK1 by ZJU-37 promotes oligodendrocyte progenitor proliferation and remyelination via NF-κB pathway
Source: Cell Death Discov. 2022 Apr 1;8:147. doi: 10.1038/s41420-022-00929-2 (PMC8975999; doi:10.1038/s41420-022-00929-2)
Supplement: Supplementary file 6 — Supplementary Table 1. [file 41420_2022_929_MOESM6_ESM.docx]

| **Supplementary Table 1** |
| --- |
| **Synthesis Process of ZJU-37** |
| 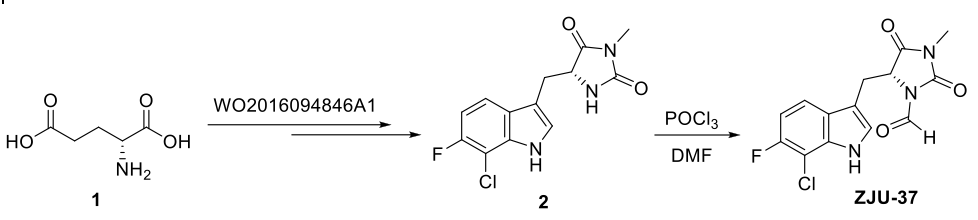 |

**Supplementary Table 1. Synthesis Process of ZJU-37.** Under the condition of nitrogen protection and ice bath, POCl3 (0.4 mmol, 60 mg) was added to N,N-Dimethylformamide (DMF) (1 ml) dropwise, and then stirred for 0.5 hours. Then the DMF solution of compound 2 (0.2 mmol, 59 mg) was added to the reaction solution, and let the reaction continued for 5 hours at room temperature. The white solid ZJU-37 was obtained by adding appropriate amount of water to quench the reaction, and was extracted with ethyl acetate for three times. The organic phase was washed with saturated salt water, and dried over anhydrous sodium sulfate. Then, the solvent was concentrated under reduced pressure, and the residue was purified by column chromatography.
