# Supplementary material for: Inhibition of RIPK1 by ZJU-37 promotes oligodendrocyte progenitor proliferation and remyelination via NF-κB pathway
Source: Cell Death Discov. 2022 Apr 1;8:147. doi: 10.1038/s41420-022-00929-2 (PMC8975999; doi:10.1038/s41420-022-00929-2)
Supplement: Supplementary file 7 — Supplementary Table 2. [file 41420_2022_929_MOESM7_ESM.docx]

| Supplementary Table 2  8 Inhibitors of Necroptosis | | | |
| --- | --- | --- | --- |
| Number | Name | Structure | **Description** |
| 4-E2 | Debriefing (GSK2118436) | 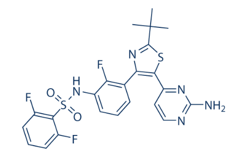 | Dabrafenib (GSK2118436) is a mutant  BRAFV600 specific inhibitor. |
| 6-F18 | Pexmetinib (ARRY-614) | 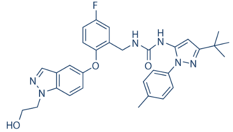 | Pexmetinib (ARRY-614) is an effective, oral  bioavailable dual p38 MAPK/Tie-2 inhibitor. |
| 6-C21 | GSK2606414 | 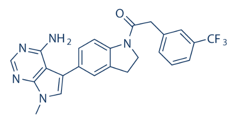 | GSK2606414 is an oral bioactive, effective,  selective PERK inhibitor |
| 5-B4 | GSK2656157 | 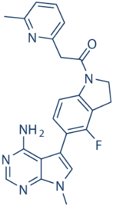 | GSK2656157 is an ATP competitive  high selective PERK inhibitor |
| 1-A3 | Nintedanib (BIBF 1120) | 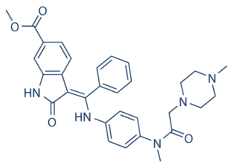 | Nintedanib is an effective triple kinase inhibitor,  which acts on VEGFR1/2/3, FGFR1/2/3 and PDGFRα/β |
| 6-I17 | TAK-632 | 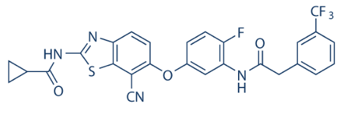 | Tak-632 is a powerful pan RAF inhibitor |
| 4-P1 | Pazopanib | 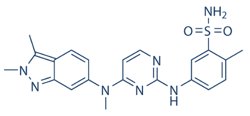 | Pazopanib is a new multi-target VEGFR1,  VEGFR2, VEGFR3, PDGFR, FGFR inhibitor |
| 3-I12 | Osthole | 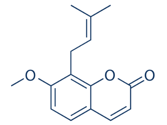 | Osthole is a coumarin compound |

**Supplementary Table 2. The top 8 compounds yielded from high-throughput screen.** The top 8 compounds were listed according to their potency on protecting Jurkat FADD^-/-^ cells from necroptosis.
