# Supplementary material for: Inhibition of RIPK1 by ZJU-37 promotes oligodendrocyte progenitor proliferation and remyelination via NF-κB pathway
Source: Cell Death Discov. 2022 Apr 1;8:147. doi: 10.1038/s41420-022-00929-2 (PMC8975999; doi:10.1038/s41420-022-00929-2)
Supplement: Supplementary file 1 — Response letter to Editorial requests [file 41420_2022_929_MOESM1_ESM.docx]

Manuscript Number: **CDDISCOVERY-21-2752R1**

Title: Inhibition of RIPK1 by ZJU-37 promotes oligodendrocyte progenitor proliferation and remyelination via NF-κB pathway

Dear Dr. Editor,

Our sincere thanks for your suggestions on revision of the present manuscript, and the suggestions were: **‘1. Please ensure all author affiliations are designated in the correct order on the manuscript title page. Please use superscript numerals after author names to connect the names to the appropriate affiliation(s). The numerals should go up in the correct order.’.**

In response, we have carefully checked the manuscript title page, and we found that in the current version, **all author affiliations are designated in the correct order using superscript numerals after author names to connect the names to the appropriate affiliations, just as what you suggested.**

We hope that the current version is now ready to be accepted for publication.

Warm regards to the editorial team for your continued help.

Sincerely yours,

Jing-Wei Zhao, PhD, MD

Professor & PI of Institute of Neuroscience, Associate Director of Cryo-EM Center, Zhejiang University, China.

E-mail: [jingweizhao@zju.edu.cn](mailto:jingweizhao@zju.edu.cn)
